# Remarks on Certain Opinions Aad Doctrines Relative to the Phagedenic Ulcer Contained in Mr. Carmichael's Late Work on Venereal Diseases

**Published:** 1819-01-01

**Authors:** G. J. Guthrie

**Affiliations:** &c. &c. &c.


### *Remarks on certain Opinions aad Doctrines relative to the Phagedenic Ulcer contained in Mr. Carmichael's late Work on Venereal Diseases.*

By G. J. Guthrie, Esq. &c. &c. &c.

THE very liberal and gentlemanly manner, with which Mr. Carmichael has noticed the observations which I and several others have made on the experiments now pursuing in the army, for the purpose of ascertaining the real nature of the venereal disease; has induced me to depart from the line of conduct I have hitherto observed, and to offer you the following remarks in elucidation of one part of the subject, on which Mr. C. and I do not entirely agree; viz. on the nature of the phagedenic ulcer. For where the object of a professional man is evidently scientific, when his inquiry is conducted in a manner as reputable to himself, as it is respectful to those whose opinions he impugns, and when the subject is of importance; a marked neglect of his observations on the part of those with whom he differs in opinion, might lead to the suspicion of a deficiency either in their love of science, or in their knowledge of good manners; or at least to a doubt of their own opinion of the validity of the arguments they support.

In the paper which I offered to the notice of the Medico-Chirurgical Society, "On the Treatment of the Venereal Disease without Mercury," and which gave rise to Mr. Carmichael's remarks in his late work, "On the Uses and Abuses of Mercury in Venereal Diseases," I was aware that the inquiry was a delicate one; that the bare mention of it would excite the irritable passions of those who were strongly prejudiced against any innovation, in what they considered a practice confirmed and established by their experience. I endeavoured, therefore, neither to lead, nor to mislead; but simply to state some facts on both sides of the question, and to give such cautions as might enable others, who had better opportunities, to continue the experiments, without incurring any danger to their own reputation, or to the health or safety of the persons entrusted to their care. Since that period the practice has been considerably extended; but so judiciously has it been conducted in the army, that in no one regiment, or hospital, has a patient, treated for the venereal disease without mercury, lost his life, been rendered incapable of further service, been mutilated by the loss of a limb, or caries of the bones of the head, or loss of the nose. The returns from the different regiments and hospitals, of the army, are most exact on these points; I have been permitted, by Sir James M'Grigor, to examine these records, and they are equally open to the inspection of any gentleman. If the results had been otherwise, I should more willingly have given them (and without delay) than I gave the successful state of the experiment; for I felt that my observations might tend to lead some few unthinking persons into error; whilst a statement, containing proofs of the inefficacy, or of the bad effects of the practice, could only tend to prevent it.

In that paper I said, I was acquainted with 500 cases of ulcers acquired from promiscuous intercourse, and which had been cured without mercury; I may now make these hundreds, thousands, proving the same fact. It is but just, at the same time, to mention, that a very few ulcers have gone on in an indolent state, or gradually increasing for several weeks, until a course of mercury was resorted to for their relief; whilst a similar course, in other instances, has increased the ulceration. The ulcers, which resisted the treatment without mercury, did not possess more marked characters of syphilis, than those which were cured without its use; and, in these instances the course was adopted rather from the consideration of time than of necessity, and by those who were not conversant in the practice; for it appears from the official reports, that in the hands of those who were confidently anti-mercurialists, no ulcer has resisted their endeavours to effect a cure without mercury; neither have they, on the average, taken up a longer time. It establishes then a fact, which, so far from being acknowledged by the most eminent surgeons in London, four years ago, was actually denied; viz. that a venereal chancre can be cured by simple applications, without mercury.

I stated, in the same paper, that the great object of inquiry was not with reference to the cure of the primary ulcer alone, but as to the recurrence of secondary symptoms. I said, that in the trials I had made, it was as one in ten; with some, it has been has high as one in three; but, in the army generally, in round numbers, as 1 in 25. To enter into these particulars, would lead us far from the object of this paper; and which, indeed, from circumstances, I am not permitted to do. I may simply state, that almost all the secondary symptoms have been of the mildest sort, (principally papular eruptions, and slight sore throats) and much more so than those which have occurred in cases, in which mercury had been used, for the cure of the primary symptoms.

The subject of discussion between Mr. Carmichael and myself, is on the specific nature of the poison producing the phagedenic and sloughing ulcers, and how far these sores are modified by the state of the constitution, including that of the part.

The different appearances found to exist in the several sores to which the sexual organs are liable, as well as the different secondary or constitutional symptoms, which followed these ulcers on many occasions, have at all times led to a classification of these diseases; or, at least, to attempts to ascertain the nature and appearance of that particular ulcer, which is more frequently the cause of these unpleasant effects; in order that the specific might be duly, and early applied to prevent them. Repeated observation had taught, that mercury, given as soon as an ulcer could be recognized to be syphilitic, would, for the most part, supersede the baneful influence of the poison on the constitution at large; and that when affected, it possessed considerable powers in restoring it to its pristine state. It was occasionally, I will even say to avoid ground for dispute, frequently found to fail, both in the cure of primary sores, and secondary symptoms; but these were then considered to be influenced by a particular state of the system, or by the obstinate primary sores, being of a different nature from tbe genuine disease.

The empire of a specific disease being once fairly established, other diseases affecting the same parts, if acquired under suspicious circumstances, were soon lost sight of, and gradually merged into varieties of the one powerful poison; or, if there were doubts, it was considered better to err on the safe side. It was, however, perpetually occurring to practitioners, to meet with negligent persons, who would not use the remedies ordered, and who got well without them; or who recovered before the remedies could be supposed to have any effect; and sometimes, while practitioners were in doubt what course to pursue, they were often surprised to see the primary sores disappear with rapidity. This led to different attempts to distinguish the one disease from all others; and several have pushed their attempts even to pointing out the particular appearance of these ulcers, which were supposed to be distinct from syphilis itself. It became necessary, then, to allow of a plurality of poisons, affecting these parts, although their origin was not so satisfactorily demonstrated; but most authors imply, that all of them may be readily generated, save the poison of syphilis, which (as of typhus fever of old, and I suspect with as much truth) was supposed to be only communicated, or generated, by contact with a person previously suffering from the same disease. The existence of a particular poison being presumed, it became necessary to account for the deviation of appearances in two ulcers situated nearly on the same parts of two males, who had acquired them from one female; and this was attributed to constitution. If one deviation were allowed to peculiarity of constitution, it was hardly possible to deny its influence to a greater extent; and thus the door was opened for what has been since considered the many varieties, the Proteus appearance of syphilis, in its primary and secondary symptoms; whether these were the actual consequences of the disease, or of the bad administration of the remedies intended for its cure. The admission of the influence of the system at large, and of the part itself, on the appearance of the ulcer, tended at once to destroy the diagnosis which had been established; and although a little might be allowed without much fear of injury, still much could not be granted, without striking at the root of all classification, which was founded on the principle, of similarity of appearance, similarity of effect. We find accordingly, that the influence of the constitution, and of the part on the appearance of the ulcer, is denied in a great measure by Mr. Carmichael, in his classification, as a primary point in his system; while he has nevertheless admitted the opinion in detail. Denying then any influence to the constitution, he is obliged to suppose an ulcer secreting the same morbid matter from its beginning to its termination; and in every stage or state, exciting, when applied to sound parts, another sore similar in appearance to itself; an opinion which, when carefully examined by fact and experiment, cannot, I conceive, be maintained; for I have reason to think that I have facts and experiment against it, as well as repeated observation. I am, then, for the present, disposed to refrain from believing in a plurality of morbid poisons, each invariably producing a disease of one distinct character; or, of one specific poison modified by the powers of the constitution, or of the part; but rather to place reliance in the combined opinion of different morbid poisons (to use the common term) being generated *in different people, and stages, and states of disease,* under circumstances which I no more understand, than others do of the nature of the syphilitic poison itself.

It is extremely difficult, in private life, to obtain any satisfactory information from patients themselves, approaching to the nature of direct experiment; thus, when I say as above, that a deviation takes place in the appearance of two sores, acquired by two men from one female; it has very seldom happened, that the sores of the three could be examined; for if it had often occurred, all the disputed questions would long since have been decided. But as these opportunities have not happened sufficiently often to the same person to make a serious impression; so when they did occur, he was little disposed to give credence to the parties, when it militated against his opinions; or if he did believe, he only considered the subject as more difficult, without endeavouring to surmount it.

Direct experiments cannot be made in this country, and few are disposed to make them on themselves. But if any of these points can be proved from circumstances almost amounting to direct experiment, few will be disposed to doubt; and I have no hesitation in saying, it is only in the army (except by accident) that circumstances can be found so sufficiently conclusive as to amount to direct experiment.

The males and females are often all under controul, the disease is ascertained to exist shortly after it is acquired; frequently the female can be pointed out, and where several men are infected from the same person, she can almost invariably be examined, as a follower: indeed, these women are sometimes received under care for the purpose of being cured. That a gonorrhœa will arise in a man, from intercourse with a female, who has no obvious disease, and shall remain free from any for weeks, no one in extensive practice will deny; we will, therefore, not admit that symptom into our consideration. But if we find one female has infected three, four, or a dozen males, within a few days; and on examination we find she has a sore, or sores, which we call syphilitic chancres, and we find the men have sores of four or five descriptions; are we to conclude she is infected by a plurality of poisons which we cannot detect; or, that the state of parts, and of the constitution generally of the males has made the difference? I am aware, that any statements of this kind drawn from books, will not be relied on; any observations of my own, made before these particular points began to be questioned, will be equally inadmissible; neither do I wish them to do more than to lead to further inquiry.

I may still be permitted to say, that I have had repeated opportunities of seeing that several, say twenty men, have acquired different kinds of sores, not excluding the phagedenic ulcer from the same female, who had only those of a common syphilitic description.

Dr. Fergusson gives a striking instance in his paper, published in the fourth volume of the Medico-Chirurgical Transactions, of a malignant affection, acquired from an opera dancer in Lisbon apparently in perfect health; and who continued upon the stage many months afterwards, occasionally infecting others, without any thing extraordinary, as far as could be observed in the nature of the symptoms. A case, perfectly analogous, occurred under my own care; and since the inquiry began, several surgeons have had so many opportunities of verifying facts of this nature, that I have no doubt this important question will soon be finally settled.

Waving then, for the present, all discussion as to the nature of other secondary symptoms, for the reason assigned; I now come direct to the question of the phagedenic ulcer. I was induced to notice Mr. Carmichael's observations on this subject in my little paper, because I thought he had drawn a greater degree of support from Dr. Fergusson's memoir, than he, Dr. F. intended, or would now be inclined to admit; and I wish to put the matter in its proper point of view, although I was aware, that if correct, it struck at the root of the opinion of a specific syphilitic phagedenic poison. I did it with the hope of attracting further attention to the subject; and should not personally have returned to it, if Mr. C. who saw the tendency of my remarks, had not endeavoured to subvert them, by doubting the correctness of the observations, on which they were founded.* A negative is as good as an affirmative opinion, where two persons only are concerned; to establish either as a fact, we must resort to others; and to Dr. Fergusson as an arbitrator, Mr. Carmichael cannot have an objection. I can only say, the ulcer in question began like all the cases of phagedena, which Mr. C. has adduced in support of his opinion; and in reply to a written question, I received the following answer from Dr. Fergusson.

"Poor E——'s case was unquestionably one of phagedenic ulcer, the result, most probably, of syphilitic innoculation on parts that had been similarly diseased, and

---

* See his late work.

been the subject of treatment innumerable times before; and in a constitution so exhausted, through antecedent mercurial courses, as to be incapable of assuming, or at least keeping up, healthy actions from any mode of treatment that could be devised." Indeed, the case is so correctly stated, that several gentlemen who saw this officer at the time, have inquired if it did not allude to Major E——.

There can be no doubt then, that it was a case of ulcer possessing all the characters of phagedena, and that peculiar character which Mr. C. has described, page 151 and 152 in his first work. "Suddenly these parts are attacked by severe pain, and afterwards assume a bluish cast, and on the following day they are found to be covered by a slough; and in this way this destructive malady continues to extend its ravages by alternate sloughing, and ulceration."

I attended a case with Mr. Morel, surgeon to the Westminster Hospital, and surgeon on the Staff at York Hospital, now Deputy Inspector, where the patient had acquired an ulcer of a simple aspect at Ostend, for which he underwent a course of mercury of six weeks duration, without any curative effect, the sore slowly increasing, without any particular pain, or characteristic appearance; but which, on his undergoing a second course, sloughed, and the glans penis was lost. Sore throat, and blotches soon followed, and he was sent to England, where he underwent another course of mercury. The remaining portion of the penis now exhibited the phagedenic character, and Mr. Morel frequently drew my attention to the edge, as the forerunner of sloughing, by reminding me of the same appearances which gave so much trouble in the case of Major E. at Lisbon. The throat and the blotches on the forehead exhibited the appearances Mr. Carmichael has described as following phagedenic ulcer, and his face was almost a counterpart to the example he has given in his last work. This man tried sarsaparilla, acids, &c. in vain, every application to the penis proved equally useless; mercury appeared at first to ameliorate every symptom on several different occasions; but when pushed to its full effect, they rapidly deteriorated. He was at last perfectly cured, and remained well to my knowledge for many months, by small doses of mercury, long continued, but never urged so as to produce a marked mercurial effect; by regular diet, air, and sarsaparilla. If cases of this kind be required, I can bring dozens of well substantiated ones, in which the ulcers were at first without any sloughing and phagedenic characters for a considerable time, yet subsequently assumed them,* and were followed by the secondary symptoms, which Mr. C. considers as the true constitutional ones of specific venereal phagedena. A reference to his own recorded cases will, I think, prove the fact; but I have other evidence. In my observation on the use of the nitro muriatic acid, published in the 48th Nnmber of the London Medical Repository, I have detailed two cases, the fourth and sixth, in which the constitutional symptoms of Mr. C's phagedenic ulcer took place. In one, the chancre did not increase so as to be troublesome to the patient, until after he had undergone a full course of mercury, having commenced, and continued for several weeks like a common sore; and it only assumed the sloughing, or phagedenic character, after he joined his regiment, and began again the use of mercury. The man is at this moment in the York hospital, where, in all probability, he will end his days. In the other case, these symptoms all followed a gonorrhœa, without any primary ulcer.

If we refer to case twenty-three, page 70, of Mr. C's last, work, we find the gentleman was disordered four months, and had undergone a course of mercury before Mr. C. saw him, who cured him by simple means in three weeks. Surely, if this had been a true primary phagedenic ulcer, it would, under improper treatment, have affected, in so long a period, more parts than the internal surface of the prepuce. In Case 26, the gentleman had been using mercury by friction for several weeks, until he was strongly under its influence, and yet the ulcer, which was increasing, had only implicated the frænum and glans to the size of a silver penny.

I have selected these cases, because they are both stated to be those of gentlemen, who have been under the care of other practitioners, before Mr. Carmichael saw them, and, therefore, in all probability, referrible to; and I do not imply any disrespect in saying, I do not agree in opinion with him, that they were primary phagedenic ulcers; but that, on the contrary, they were common ulcers which subsequently became so; and the fact can only be verified

---

* The Editor of this Journal lately attended an officer who had, for five weeks, been affected with a common looking chancre on the glans penis, and had been all that time using mercury moderately. All at once, and without ostensible cause, it put on the phagedenic appearance, and the glans penis was lost in a week. Extreme depletion, starvation, and opium prevented the penis also from going. EDIT.

by the gentleman under whose care both were, for so long a time.

Pursuing our researches in the same manner; if we turn to Case 32, we find an ulcer had existed about two months, for which the patient had taken twenty pills, and rubbed in two ounces of mercurial ointment, so as to affect his mouth; but which ulcer was "about the size of a sixpence, exhibiting a white sloughy appearance, situated on the body of the penis, the integuments of which were undermined to some little extent," and which subsequently burrowed under the skin. Can we allow this to be a case of true primary phagedena, according to the description laid down? and how can we account for the mildness of the symptoms, but by supposing that it was not a primary phagedenic ulcer; or, that the constitution had modified or altered the course of the disease, it being so unlike other cases of phagedenic constitutional affection?

I consider Case 34, as no less conclusive. The patient was admitted on the 20th of September, "with a phagedenic ulcer of the prepuce, a great part of which had been destroyed by its ravages; a bubo on the right groin, pains in his joints, and his mouth severely affected by mercury, which he stated he had been using, from the appearance of the ulcer, a period of five months." Two months after, (the 24th of November) it is stated, that the ulcer had destroyed a considerable part of both the glans and body of the penis. Here then, under the worst possible treatment, during five months, we find a phagedenic ulcer only destroys a great part of the prepuce; whilst in two months, under the best, it destroys a considerable part of the glans and of the prepuce; and, because it is followed by certain constitutional symptoms, it is declared to have been a primary phagedenic ulcer. I am well aware of the difference which exists between curing an ulcer at an early, and at a late period; but I think I have still fair grounds for supposing this ulcer, in the first instance, to have been a simple chancre, and not a phagedenic one, and that it only became so from long continued bad treatment. It rather appears to me, that the inference is not on a better foundation than the one formerly entertained, and which is now so much reprobated, "that whatever secondary symptoms mercury will cure, must consequently have been venereal; and that those it will not cure, are in all probability otherwise."

Case 35, James Carroll, admitted April 27, 1815; "an extensive ulcer of the glans surrounded the orifice of the urethra. Ulcerated bubo in the right groin. He stated he had been four months disordered, during the whole of which period he had been using mercury, which kept his mouth continually sore. Solut. antim. catapl. During the first week in May, several spots, covered with crusts, had appeared on his back, shoulders, and forehead. The ulcer on the penis had become more irritable, on which account the antimonial solution was discontinued for five grains of extract of cicuta, three times a-day." Surely there is even stronger reason for believing this to have been at first a case of common ulcer.

Case 46, is still more strong. I have here confined myself to the cases related in the last publication; but if the first be referred to, there are several liable to the same objections; and proving, so far as the evidence goes, that ulcers, after existing in a simple state for some time, take on occasionally phagedenic characters; and are then sometimes followed by such secondary symptoms as Mr. C. has described as only succeeding the specific phagedenic ulcer.

Every man has a right to profit by his experience, and society at large is obliged to him for detailing it; and, by recording his errors, his successes, and his observations, establishing, as it were, a beacon to guide others out of the labyrinth in which they are involved. Mr. Carmichael has done this in an eminent degree; but in doing it in his present work, in relation to phagedenic ulcer, he has advanced some new doctrine, which materially alters the state of the argument, with reference to that part of the former one, which was at variance with my experience. In the first publication, he appears to consider all phagedenic and sloughing ulcers of the penis, possessing the characters he describes, as dependent on the same specific poison, and followed by the same constitutional symptoms: which is the opinion I combated in my paper. There is no reservation that I have observed, as to common ulcers, which have, after a time, become phagedenic or sloughing, not causing the same appearances; but, on the contrary, a very different set of symptoms; which is the theory now advanced in the second book.

In the period which elapsed between his first and second publication, further experience proved to Mr. C. that his opinions were not well founded, in regard to these latter sores; and he has made them the subject of a particular exception, to avoid invalidating the general doctrine, which will be found at page 8, to which I refer, and again at page 61, as follows: "It is true, that neglect, local irritation, and I will even add (although it may seem to favour the opinion of my adversaries) excessive constitutional irritability, will cause a venereal ulcer, as it will any other, to become phagedenic, however originally mild in its nature. It must, therefore, be in the highest degree useful to attend to the progress of an ulcer, and, if possible, to ascertain whether it was of the phagedenic species from its commencement or not; and if not, it is not to be classed with that venereal disease, which may be termed phagedenic. That the distinctions I have here pointed out are worthy of attention, will be admitted by those who are acquainted with the circumstance, that a difference will manifest itself between the constitutional disease arising from the one and the other; instances of which will be found in the cases detailed in the course of the work.—*That* of the truly phagedenic species will be characterised by pustules or tubercles, which, almost immediately from their commencement, form ulcers covered with crusts; and a train of constitutional symptoms follow, which correspond with the eruption in obstinacy and malignity: while that of the ulcer, which has only become phagedenic from irritation, &c. will display a scaly or papular eruption, according to the nature of the primary ulcer, at its commencement."

This definition of the sloughing ulcer, if it is to be *strictly observed,* also puts an end to the argument; for what the secondary symptoms may be, following such a sore, I really do not know: and taking the Case, No. 68, of the first publication, as a genuine example of it, I honestly declare, I cannot recollect more than two cases, any way resembling it, having occurred to me, out of several thousand instances; I do not believe that the proportion of such cases is greater than one in five thousand.

I have no hesitation in saying, no analogous disease (if it be one) existed in Portugal at any period whilst the British Army was in it;* and therefore, Mr. Carmichael's opinion most certainly receives no support from any thing that occurred in that country. On the contrary, our experience was decidedly at varience with him; for the symptoms he asserts to be peculiar to the specific phagedenic ulcer, and the specific sloughing ulcer; did, I affirm, frequently follow the common ulcer of both de-

* I am authorized by Mr. West, Deputy Inspector of Hospitals attached to the Portuguese Army, who has lately arrived from Lisbon, to say, no such disease has been observed since, either in the civil or military hospitals.

scriptions; and until Mr. Carmichael shall bring proof, either from his own observation, or that of other respectable men, that the ulcers he alludes to, and which I have noticed above, as existing for weeks, and months, before he saw them, were phagedenic or sloughing from their commencement; I cannot bring myself to believe, that several of them were any thing more than common ulcers becoming phagedenic, from neglect, ill treatment, &c.; and therefore, according to his last definition, out of the pale of his constitutional symptoms.

In this opinion, I suspect, most impartial men will coincide; and so far from adding strength to his doctrine by his last publication, he has, I think, sapped the foundation on which it was erected; inasmuch as I conceive the cases he relates, prove the reverse (in their present state) of what they were intended to establish.

Referring to Mr. Carmichael's first work, page 124, we have a sketch of the progress of the phagedenic ulcer; but we do not find any description of that ulcer, from the commencement of ulcerative absorption, as we do of the sloughing ulcer, from the commencement of the sloughing process. P. 151. Neither is one given in the second book; so that we are left in doubt whether his specific phagedena begins as such, or, whether it assumes that character after a certain lapse of time. If the former be the fact, it adds strength to my objections to his cases; if the latter, it is necessary we should be informed of the precise period required for the specific character to show itself: for if small-pox (the received laws of which he frequently alludes to in illustration of his opinion) be a fair analogy at one stage of the disease, it must be equally so at another; and a definite period can, and ought to be, assigned for the appearance of the specific character after the inoculated part has begun to ulcerate. Again, if the constitution in general, and of the part in particular, have little or nothing to do with the appearance or nature of the ulcer; how is it that there is so marked a difference in the appearance and progress of a phagedenic and sloughing ulcer? If, as Mr. C. supposes, page 121, first work, "the constitutional symptoms also of both species are much of the same character, so that it is likely that they also arise from one and the same virus." For the difference between a phagedenic and a sloughing ulcer, is as great as can be desired between two ulcers of different kinds, so long as the latter preserves its sloughing character: when it changes, and assumes the phagedenic character, I admit the resemblance; but I see no way in which the great difference during the first and characteristic stage can be accounted for, except by referring it to the state of the constitution in general, and of the part in particular. If these can cause so marked a difference in one instance, why not in another: and what becomes of our diagnosis? In the same manner, in giving a description of chancre, we find as great a difference between the characters of a syphilitic ulcer on the glans penis, and on the body of the penis, as can well be required by the most fastidious classifier of ulcers, from external appearances. P. 25, et seq.

"The primary syphilitic ulcer has been admirably well described by Mr. Hunter, in the following words: 'The sore is somewhat of a circular form, excavated, without granulations, with matter adhering to the surface, and with a thickened edge and base. This hardness or thickening is very circumscribed, not diffusing itself gradually and imperceptibly into the surrounding parts, but terminating rather abruptly."

"Chancre, when situated on the body of the penis, is of a dark livid colour; the ulcer is not excavated, but is on a level with the surrounding parts. It is attended with less induration than the excavated chancre; and is, in general, from the size of a sixpence to that of half-a-crown; and even, sometimes, it extends round the body of the penis. Its edges are a little raised, and the surrounding induration very perceptible to the touch, though not in so great a degree as the chancre described by Hunter.

"If mercury be employed, the ulcer soon assumes a healthy appearance; but if that medicine be not resorted to, its livid surface is alternated every third or fourth day with that of light brown or tawny colour, the ulcer at the same time extending its dimensions slowly; and as it advances, the surrounding induration obviously increases."

If it be not the texture of the part that causes the difference, what can it be? If we allow that difference of texture can cause a difference in the appearance of a specific ulcer, we must allow that these ulcers follow the common law of ulceration in general; and that a healthy or unhealthy state of the texture will make a difference in the character of the ulcer. It is a position that cannot, in my opinion, be controverted; and goes a great way to overturn our faith in the axiom which Mr. C. would inculcate, of specific virus, specific character under every circumstance. If the line be drawn so distinctly between a primary phagedenic and sloughing ulcer, and a consecutive one, or an ulcer on which either has supervened, what becomes of the assistance to our diagnosis, to be drawn, as he recommends, page 106-7, from the loss of the penis, or the cicatrix of the primary ulcer?—It is absolutely nugatory—the same loss or appearance follows the one kind of ulcer as the other; and I can now show a man, with every symptom that was present in the particular case on which this observation is recorded, (John Martin, p. 166.) in whom the primary ulcer was not phagedenic, although it subsequently became so. I had another lately under my care, who has recovered without any mutilation, in whom the symptoms were so nearly alike, that many thought his nose would be lost; yet the mark of the primary sore was hardly observable, and the ulcer itself healed in a very short time, under the administration of a few pills, which did not affect his mouth.

If the objections which I entertained to Mr. C's opinion, as expressed in his first work, were removed; the further alteration and addition he has made to his doctrine in the second, would still require elucidation. In the first, we find, that the tubercular eruption is considered as a distinct constitutional symptom, and arranged as the fourth in order, page 216; and he was then disposed to attribute it to the burrowing ulcer. In the second work, we find he distinctly classes the pustular and tubercular eruptions under one head (page 62), and refers them to the phagedenic and sloughing ulcer. At page 155, he details two cases of tubercular eruption, referring to plate 2d of his former work to insure greater accuracy, and yet allows that one did not follow any kind of ulcer; and, that the ulcer in the other was not phagedenic or sloughing, is sufficiently evident. If we refer to the first work, we find cases, and especially the 83d, in which there were no primary ulcers; and, as if further to overturn his own theory, he immediately, in his second book, follows these cases up by the relation of others equally objectionable, and particularly the following, Case 63.

"On the 12th of June, 1815, I was called upon to see a young unmarried lady, whose condition and morals placed her altogether beyond the reach of suspicion; yet the symptoms with which she was affected *precisely* resembled those which are undoubtedly of venereal origin. There was a considerable number of large tubercles similar to those described in the last chapter, scattered over her legs, arms, and thighs, attended with discolouration of the integuments. She complained of soreness of her throat; and on examination, I found the back of the pharynx ulcerated, and covered with white tenacious matter, evincing a similar correspondence between the affection of the skin and throat, to that which I had often witnessed in venereal cases. Her tongue was white and furred, with bad appetite and general derangement of the system.

"I merely regulated her diet, ordered five grains of blue pill every night, and three drachms of sulphat of magnesia every morning; under which plan she had perfectly recovered before the 12th of July following, when I ceased to visit her.

"I might adduce many instances of *similar* tubercles terminating in ulceration in children, in whom they are generally esteemed to be signs of a strongly marked scrofulous constitution, and cannot be suspected to be venereal complaints, because they occur too late after birth to be ascribed to a venereal taint, derived from the parents; and too early in life, to be visited as an imputation on the young patients themselves. But by whatever name the disease may be called, it is in children, as well as in adults, accompanied by general derangement of the constitution; and yields in both to the same mode of treatment, viz. small alterative does of mercury, conjoined with decoction of sarsaparilla."

If these cases did occur without any specific primary phagedenic or sloughing ulcer, they must be attributed, as he truly attributes the last, to a particular state of the constitution; and if they can occur, so as not to be distinguished from any others, but from the history of the case, and other concomitant circumstances; how can they be supposed to be dependant on a particular ulcer, and to follow it as cause and effect? If a constitutional derangement, unattended by a particular ulcer of the genitals, can cause a tubercular eruption, or other symptoms; I do not think the circumstance of the addition of the particular ulcer, a sufficient reason for believing the state of the constitution has no influence in producing the very symptoms it is capable of giving rise to, without the ulcer.

In addition to the pustule and tubercle being now considered as joint symptoms of the same disease, a new character is added to both, not marked in the former work as distinctive, viz. the ulcer healing from the centre instead of from the edges, vide pages 10-158. Yet, so far from these being truly distinctive characters, if we refer to page 144 of the first work, Case 59, we find an eruption, both papular and pustular, existing at the same time.

At page 167, Case 70 is given as one of successful treatment of sloughing ulcer; and we find it is stated at page

169, that on the 8th of September, the constitutional ulcers on "the arm and forehead were clean, granulating, and healing round their edges." In these two cases then, which, as to matter of fact, must remain undisputed, although the inferences to be deduced from them may be altered; we have it recorded, that the constitutional eruption of phagedenic ulcer may be not only pustular, but papular, and even resembling pimples, some containing a little matter at their apex; and that the constitutional ulcer following the sloughing ulcer, can, and does heal at its circumference. For if the ulcers in this case had healed from the centre, and not from the circumference; this circumstance could hardly have escaped so close an observer as one, who alludes to the particular manner of their healing, when it is the usual way in which ulcers in general heal; and had, therefore, nothing extraordinary in it.

I am aware that many people, on reading over these two cases attentively, may suppose that neither of them were true cases of primary specific phagedena, or slough; but, that they were common ulcers, irritated by irregular or improper treatment, or of another description; and this is the light in which I view them. But this weakens rather than supports the doctrine; for, it proves what Mr. Carmichael is most desirous to disprove, *that pustules and tubercles do occur as a constitutional symptom of common ulcers, which have become phagedenic, or sloughing*; and shews, that when I said, "that in a constitution where an ulcer rapidly became phagedenic, the secondary symptoms, when they do occur, may be different to a certain extent from those that follow more simple ulcers in a healthier habit of body," I did not, as he is disposed to think, endeavour to make my preconceptions harmonize with my experience; but, on the contrary, I endeavoured to found my opinions on the basis even of his recorded observations.

The difference of opinion which exists between us, does not, I am happy to say, extend to the mode of curing these complaints, except in one instance. In the antiphlogistic treatment of the phagedenic and common sloughing ulcer, I fully coincide; it is the practice which Dr. Fergusson recommended before Mr. Carmichael wrote, and which I have for some years pursued. I concur with him in the necessity of abstaining from the use of mercury in the earlier stage of the constitutional symptoms, whilst I bear the strongest testimony to its great utility in the latter, when administered with caution.

In the ulcer which Mr. Carmichael describes as a primary specific sloughing ulcer, and which I have said does not occur in one case in five thousand, I certainly should not adopt his plan of blood-letting; for where a sloughing ulcer begins by a gangrenous spot, without pain or surrounding inflammation; it appears to me to partake more of the nature of a disease of the same appearance, which arises from the bad quality of the food the patients have subsisted on, or, to the gangrenous toes of old people, and of bad habits "of great eaters (as Mr. Pott says), rather than of great drinkers," than to the true phlegmonous gangrene; which, in the first stage, admits of, and is relieved by, blood-letting.

I have not noticed what I consider other material changes in Mr. Carmichael's opinions since he wrote his first book, and even since he began his second; because I believe such chastening correction as his works require, cannot be in better hands than his own; it is not my intention to impugn his opinions, but to defend mine. One point only I must not omit; it is the displacing of the ulcer without induration, but with elevated edges, from its situation in the first class of diseases resembling syphilis; and now making it hold an intermediate place between the first and second; and having for its constitutional eruption a pustule nearly resembling that of the phagedenic ulcer.

I do not object to this arrangement, which adds a link which was wanting in the chain; but I mention it now, as I think I foresee it may hereafter become a refuge to many in distress, and open a door for new opinions, and further discussions.

Mr. Carmichael, in supposing I am a supporter of the indiscriminate use of mercury, is mistaken; no one has done more, both in private conversations and in my lectures, than I have done, to put the subject in a proper point of view, and to carry on in the army, under due restrictions, the experiment of treating all venereal diseases without mercury. I have done this from the firm conviction that it is by such proceeding alone, we shall ever arrive at any thing like a perfect knowledge of the disease; of its rise, progress and termination, with the mode of cure best adapted to all stages of disease and all kinds of constitutions. It is only from the reports of surgeons of regiments we can form accurate conclusions as to the specific nature of the disease; whether it be an individual poison, modified by concentration of virus, or altered by the texture and state of the part; or, whether there be a plurality of poisons capable of producing a variety of symptoms, together with the peculiar nature of each. In making these observations, I wish the facts I have stated as to the non-occurrence of secondary symptoms in regimental hospitals, where mercury has been used, to be kept in view; and it is essentially necessary that the precautions I have recommended in the latter part of the paper be strictly adhered to, where venereal ulcers are attempted to be cured without mercury. At the same time, I am desirous that the value of mercury as a remedy shall not be deteriorated, that it shall be used, when necessary, but not abused; for if there be a man so absurd, or rather, so enthusiastic, as to suppose it can be dispensed with in every case; I can only regret, for the sake of his patients, his aberration from sound practice.

I sincerely hope I have not mistated any of Mr. Carmichael's opinions, and I cannot find a single point on which I feel I have been remiss; if there be one, it is perhaps in not giving him sufficient credit, for such allowances in favour of difference of constitution, as he is disposed to concede. At page 217 of the first Book, and page 8 of the second, the most important may be met with. In concluding my remarks on these points which have given rise to a difference of opinion, I wish it to be distinctly understood, that if there be one word that can possibly give pain, or excite even a disagreeable, although momentary feeling, it has escaped my observation, or it should have been erased; for I have too much respect for Mr. C's labours, and too high an admiration for the zeal and ability with which they have been conducted, to offer him the slightest intentional offence.

